# Detection of Cytomegalovirus in Chorionic Villi for the Prenatal Diagnosis of Congenital Infection: A Systematic Review of Diagnostic Test Accuracy

**DOI:** 10.3390/medicina62101936

**Published:** 2026-10-08

**Authors:** Ilenia Mappa, Francesco D’Antonio, Paola Ciolli, Martina Derme, Giuseppe Maria Maruotti, Giuseppe Rizzo

**Affiliations:** 1 Department of Mother and Child Health and Urological Sciences, Sapienza University of Rome, Policlinico Umberto I, Viale del Policlinico 155, 00161 Rome, Italy; ilenia.mappa@uniroma1.it (I.M.); paola.ciolli@uniroma1.it (P.C.); martina.derme@uniroma1.it (M.D.); 2 Department of Obstetrics and Gynecology, Gabriele d’Annunzio University, Via dei Vestini 31, 66100 Chieti, Italy; francesco.dantonio@unich.it; 3 Department of Neuroscience, Reproductive Sciences and Dentistry, University of Naples Federico II, Via Sergio Pansini 5, 80131 Naples, Italy; giuseppemaria.maruotti@unina.it

**Keywords:** cytomegalovirus, chorionic villus sampling, prenatal diagnosis, diagnostic accuracy, systematic review, congenital infection, QUADAS-2

## Abstract

*Background and Objectives:* Congenital cytomegalovirus (CMV) infection following primary maternal infection in the first trimester carries the highest risk of long-term sequelae, yet prenatal diagnosis depends on amniotic fluid polymerase chain reaction (PCR), which cannot be performed before 17 weeks. Detection of CMV in chorionic villi at 11–14 weeks has been proposed to bring this information forward and to exclude CMV-related embryopathy. We systematically reviewed the published studies that have evaluated this test in order to determine its diagnostic accuracy. *Materials and Methods:* We conducted a systematic review of diagnostic test accuracy (PROSPERO CRD420261482510), and reported according to PRISMA-DTA. Five databases were searched up to 19 August 2026 without language or date restrictions. Eligible studies enrolled pregnancies with documented primary maternal infection and allowed reconstruction of a two-by-two table against CMV DNA in neonatal urine and/or saliva within three weeks of birth (with fetal or placental tissue accepted for pregnancies ending in termination). Screening, extraction and QUADAS-2 assessment were performed in duplicate and blinded. Overlapping cohorts were reduced to the largest, most completely verified report. Wilson score confidence intervals (CIs) were used; pooling was pre-specified only for four or more independent datasets. *Results:* Of 704 unique records, three independent cohorts in three primary publications were eligible, contributing 340 villus samples. The largest cohort (*n* = 330) gave a sensitivity of 40.5% (95% CI 27.0–55.5), specificity of 99.3% (97.5–99.8), positive predictive value of 89.5%, and negative predictive value of 92.0% at a prevalence of 12.7%. The remaining cohorts contributed four and six samples in turn, all true negatives. No study was at low risk of bias in all four QUADAS-2 domains; the cohort carrying the sensitivity estimate was at high risk in two, including verification of terminated pregnancies on placental tissue, whose net effect on sensitivity cannot be determined from the published data. Certainty was very low throughout. *Conclusions:* CMV PCR on chorionic villi is highly specific but misses approximately three infected fetuses in five, so a negative result does not reliably exclude congenital infection, and the proposition that it excludes embryopathy is unevaluated. Based on current evidence, it should not be used as a standalone basis for first-trimester decisions about continuing a pregnancy.

## 1. Introduction

Congenital cytomegalovirus (CMV) infection is the most common congenital infection and the leading non-genetic cause of sensorineural hearing loss [1]. Its consequences depend sharply on timing; long-term sequelae after maternal primary infection are essentially confined to infections acquired in the first trimester or periconceptionally, and roughly one third of fetuses infected in that window develop sensorineural or neurological impairment [2]. Even among infected fetuses the outcome is heterogeneous, and adverse perinatal outcome is predicted by amniotic fluid viral load and prenatal imaging rather than by infection status alone [3,4].

Amniotic fluid polymerase chain reaction (PCR), the established prenatal test, is reliable only from 17 weeks of gestation onward and at least eight weeks after maternal infection [5]. This creates a diagnostic gap precisely where the clinical stakes are highest. A woman whose seroconversion is identified at 10 or 11 weeks therefore faces several weeks in which no fetal diagnostic information is available, at a gestational age when termination is legally and technically simplest. High-dose valaciclovir reduces vertical transmission after early primary infection and has changed what these weeks are used for, but it does not close the diagnostic gap [6,7].

Chorionic villi are the tissue through which the virus reaches the fetus, and they are already sampled routinely at 11 to 14 weeks for cytogenetic indications. Direct detection of viral DNA in villi has accordingly been proposed as a first-trimester alternative, with the explicit aim of excluding CMV-related embryopathy and thereby sparing women a decision taken without information [8]. Individual cohorts have been published over three decades, most recently with a large series from a single national referral centre, but the evidence has never been appraised systematically, and reports differ in era, assay, gestational age at sampling, and reference standard.

We therefore conducted a systematic review of the accuracy of direct CMV detection in chorionic villi for the diagnosis of congenital infection after maternal primary infection in early pregnancy.

## 2. Materials and Methods

The review was registered prospectively (PROSPERO CRD420261482510) and is reported according to PRISMA-DTA [9] (Supplementary Table S1).

### 2.1. Eligibility Criteria

We included studies of pregnancies with virologically documented primary maternal CMV infection with a dated seroconversion window, in which CMV was sought directly in chorionic villi obtained by chorionic villus sampling (CVS) in an ongoing pregnancy, irrespective of gestational age and sampling route, so that transabdominal placental biopsies performed in the second trimester (late CVS) were eligible whereas villi obtained at termination of pregnancy were not (E3), and in which enough information was reported to reconstruct a two-by-two table against an eligible reference standard. The primary reference standard was CMV DNA or virus in neonatal urine and/or saliva within three weeks of birth; for pregnancies not ending in live birth, verification on fetal or placental tissue was accepted as reported by the source study, and its consequences were assessed with QUADAS-2 (Section 2.5)—amniotic fluid PCR was accepted as a secondary reference standard, analyzed separately, and never pooled with the primary. Ten exclusion categories were pre-specified (E1 to E10), one assigned per record hierarchically, with companion publications (E9) taking precedence. Studies contributing fewer than four villus samples were excluded (E6).

### 2.2. Search Strategy

MEDLINE via PubMed, Embase, Scopus, Web of Science, and Cochrane CENTRAL were searched on 19 August 2026 without language or date restriction. Records were deduplicated in Rayyan [10]. The protocol committed to external peer review of the search strategy by an information specialist (PRESS); this was not undertaken, because no information specialist was available, and is recorded as a deviation from protocol. To examine its consequences, control searches were run in PubMed by adding further candidate terms (CVS[tiab]; “placental tissue”[tiab]; both together) and a known-item check on the included and companion reports; none retrieved a record that the final strategy had missed, so the deviation is unlikely to have reduced the yield. Full search strategies are given in Supplementary Table S2.

### 2.3. Study Selection and Data Extraction

Titles, abstracts, and full texts were screened independently and blinded by two reviewers, with disagreements resolved by a third. Data were extracted in duplicate into a piloted spreadsheet (Supplementary Table S3). Where two-by-two counts were not presented directly they were reconstructed from reported numerators, denominators and accuracy statistics, and cross-checked against the text; in one included report, the abstract misattributes to the prediction of congenital infection—a table that in fact describes agreement with amniotic fluid—and extraction was therefore performed from the text’s results and tables only.

### 2.4. Handling of Overlapping Cohorts

Where several reports described overlapping populations, the report with the largest sample and most complete verification was retained with the others treated as companion publications contributing no two-by-two data. Enrolment windows and sample sizes were used to reconstruct the nesting, which is set out in Supplementary Table S4.

### 2.5. Risk-of-Bias Assessment

Two reviewers independently applied QUADAS-2 [11], with signalling questions tailored to the review question, including items on whether verification of pregnancies not reaching live birth was pre-defined and on the material used, as well as on antiviral treatment received between the index test and the reference standard. Disagreements were resolved by a third reviewer. QUADAS-C was not applicable as no study compared two or more index tests in the same participants. Tailored signalling questions are given in Supplementary Table S5.

### 2.6. Statistical Analysis

Sensitivity, specificity, and predictive values were calculated with Wilson score confidence intervals (CIs). The protocol pre-specified that bivariate or hierarchical summary receiver operating characteristic modelling would be undertaken only with four or more independent datasets; with two or three, sensitivity and specificity are described separately by cohort. Deeks’ test requires ten or more datasets and was not performed. In such an event, the meta-analysis is not merely precluded by this rule but is infeasible for inclusion. Only one dataset contributed information on sensitivity, and the bivariate model requires both sensitivity and specificity in every contributing study, which is undefined in the two cohorts that contained no infected pregnancy. Univariate pooling of the three specificity estimates was calculable but uninformative, since 288 of the 298 uninfected pregnancies were derived from one cohort and heterogeneity cannot be estimated from three studies. The certainty of evidence was assessed with GRADE adapted for test accuracy, separately for sensitivity and specificity [12].

Because only one cohort contained infected pregnancies, its estimates were used, without pooling, to express the results for a hypothetical reference population of 1000 women with first-trimester primary infection (Table 1). The pre-test probability was set at the prevalence of congenital infection observed in this cohort (42/330, 12.7%), in which all women received valaciclovir. The number of infected fetuses (127) was multiplied by sensitivity to obtain true positives and the number of uninfected fetuses (873) by 1 − specificity to obtain false positives; the remaining cells were obtained by subtraction, and ranges were obtained by repeating the calculation at the limits of the 95% CI. The post-test probability of infection after a negative result was calculated with Bayes’ theorem as prevalence × (1 − sensitivity)/[prevalence × (1 − sensitivity) + (1 − prevalence) × specificity], which equals 1 − negative predictive value. These figures are rescaled as single-study estimates rather than as a synthesis, and they assume that the accuracy observed in one treated cohort applies at the stated prevalence; to illustrate their dependence on prevalence, the calculation was repeated at a prevalence of 35%, approximating the expectations without antiviral prophylaxis (Section 4.2).

## 3. Results

### 3.1. Study Selection

**Figure 1:**
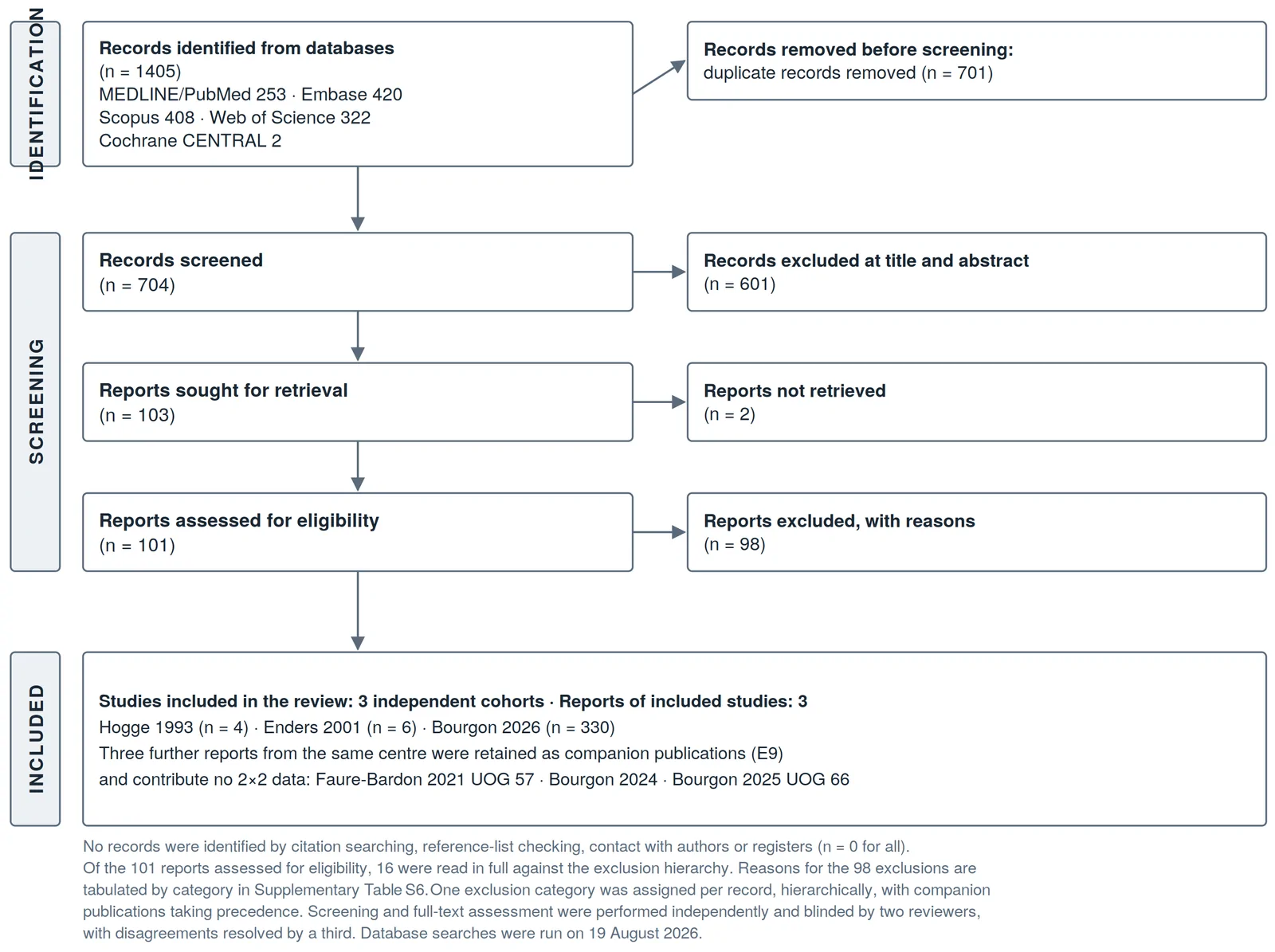
Study selection (PRISMA 2020 flow diagram [13]). Reasons for the 98 full-text exclusions are given by category in Supplementary Table S6.

The searches returned 1405 records, of which 701 were duplicates, leaving 704 unique records. After title and abstract screening, 103 reports were sought for retrieval, two were not retrieved, and the full texts of the remaining 101 were assessed for eligibility. In 85 of these, ineligibility was evident on first inspection of the full text; the other 16 required detailed reading of the methods and results, after which 13 were excluded and three included. In total, therefore, 98 reports were excluded following full-text assessment, as shown in Figure 1; the reasons why are given by category in Supplementary Table S6. Three studies were included (Figure 1). No records were identified by citation searching or other methods.

### 3.2. Study Characteristics

The three cohorts [14,15,16] are summarized in Table 1. They contribute 340 villus samples in total, of which 330 come from a single national referral centre. The two older series are small and heterogeneous: in one, only one out of four samples was a true first-trimester transcervical procedure, with the remainder being second-trimester transabdominal placental biopsies which sampled the same tissue and met the eligibility criteria (Section 2.1)—excluding them would leave a single sample, below the E6 threshold, and would remove four true negatives without altering any estimate from the largest cohort; in the other, villi were available in only six out of 189 pregnancies with known outcomes, and neither the risk group nor per-case data were reported. Three further reports from the largest centre were retained as companion publications [8,17,18]: their enrolment windows are nested within the primary report, and presenting them independently would have inflated the apparent evidence base fourfold (Supplementary Table S4).

**Table 1:** Characteristics of the included studies.

	Hogge 1993 [14]	Enders 2001 [15]	Bourgon 2026 [16]
Setting	United States	Stuttgart, Germany	Necker, Paris (national referral centre)
Enrolment period	Not stated	1988–2000	Oct 2019–Dec 2024
Parent cohort	8 pregnancies	189 pregnancies with known outcomes	330 with CVS and amniocentesis
Villus samples	4	6	330
Gestational age at sampling	10–16 weeks	10–18 weeks	11–14 weeks
Sampling route	1 transcervical, 3 transabdominal	Not stated	Transabdominal CVS
Index test	PCR on villi	PCR and virus isolation in culture	Quantitative CMV PCR on villi
Reference standard	Neonatal urine culture	Neonatal urine within 2 weeks	Neonatal urine and/or saliva within 3 weeks; placental tissue after termination
Antiviral exposure	None	None	Valaciclovir systematic throughout

CVS, chorionic villus sampling; PCR, polymerase chain reaction.

### 3.3. Diagnostic Accuracy

Against the primary reference standard (Table 2)—neonatal urine and/or saliva for live births and, in the largest cohort, placental tissue for pregnancies ending in termination—the largest cohort reported a sensitivity of 40.5% (95% CI 27.0–55.5) and a specificity of 99.3% (97.5–99.8) at a prevalence of congenital infection of 12.7%; positive and negative predictive values were 89.5% and 92.0%. The remaining cohorts contributed four and six samples in turn, all true negatives, giving specificities of 100% and confidence intervals extending to 51.0% and 61.0%, respectively, but with no information on sensitivity. Against amniotic fluid as a secondary reference standard, sensitivity was 48.3% in the primary report and 50.0% and 53.3% in the two companion reports (Table 3). No pooled estimate was calculated. Table 4 rescales the estimates of the largest cohort, the only one contributing infected pregnancies, to a hypothetical population of 1000 women tested at the observed prevalence (Section 2.6). On these assumptions, a negative result lowers the probability of congenital infection from 12.7% before the test to 8.0% (ranging from 6.1% to 9.7% across the 95% CI of sensitivity).

### 3.4. Risk of Bias

**Figure 2:**
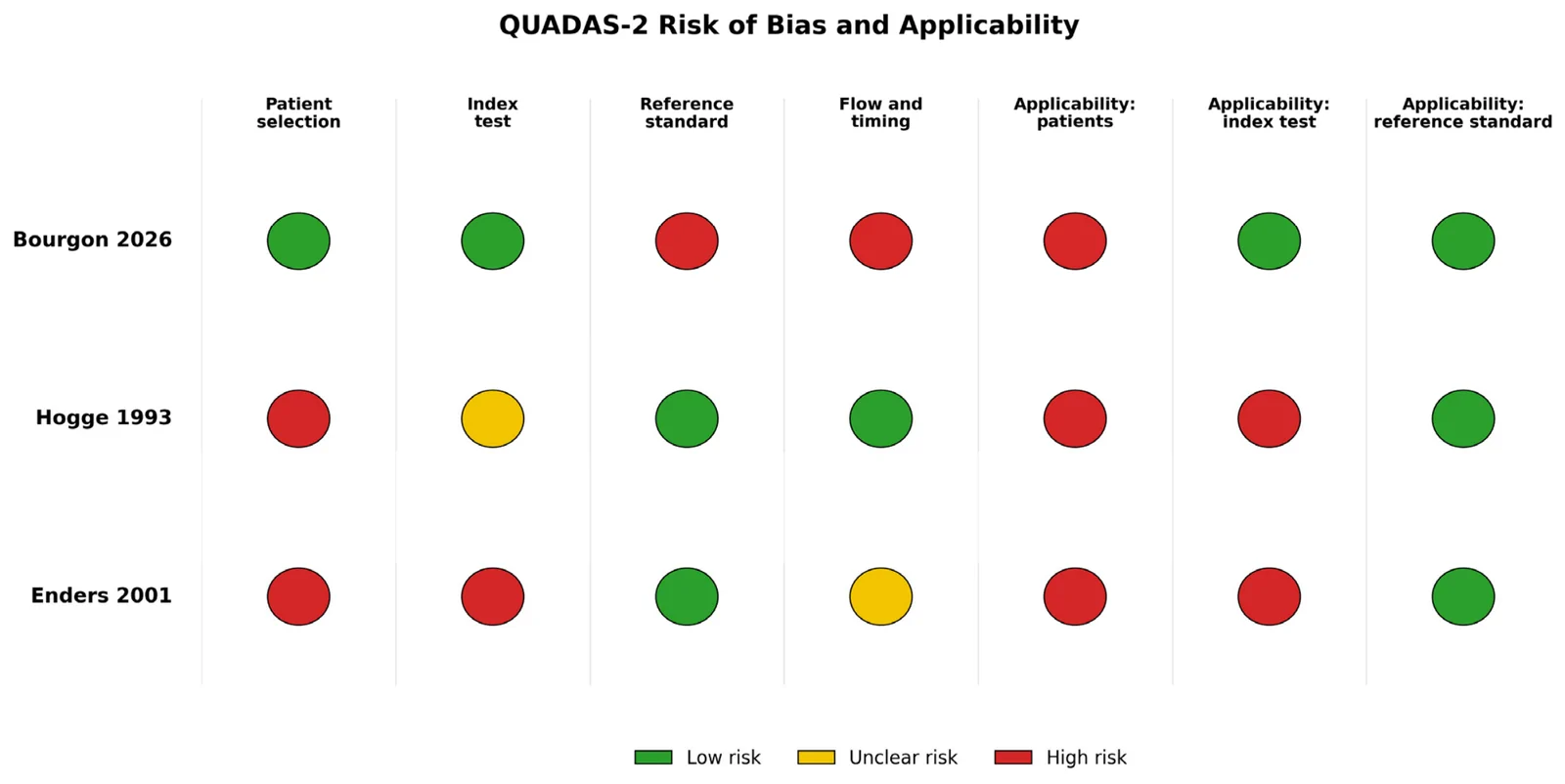
Risk of bias and applicability of the included studies, assessed with QUADAS-2 [11]. Green denotes a low risk, yellow as an unclear risk, and red as a high risk [14,15,16].

No study was at a low risk of bias across all four domains (Figure 2). The largest study, which alone carries the sensitivity estimate, was at low risk for patient selection and the index test but at high risk in two domains. Antiviral prophylaxis was administered systematically from the time of serological diagnosis up to and throughout the enrolment period; that is, in the interval between the index test and the reference standard. Pregnancies ending in termination were retained in the denominator and verified—which removes partial verification—but were classified using placental tissue. The two older series were at high risk for patient selection, with villus samples having been available in small and undocumented subsets of their parent cohorts, and raised high applicability concerns for the index test; their assays were not comparable to contemporary quantitative PCR.

### 3.5. Certainty of Evidence

Certainty was very low for sensitivity and downgraded for risk of bias, indirectness arising from universal antiviral prophylaxis, and imprecision. Certainty was very low for specificity, and downgraded for risk of bias and indirectness; the ten samples from the two older cohorts are not affected by the verification problem described in Section 4.3 but are too few to sustain a higher rating.

## 4. Discussion

Direct detection of CMV in chorionic villi from 11 to 14 weeks is highly specific and insensitive, missing approximately three infected fetuses in five. The asymmetry that matters clinically is that specificity is estimable, within limits, from three centres and three eras, whereas sensitivity rests on 42 infected pregnancies studied in one laboratory.

### 4.1. Reassurance About Infection Is Not Reassurance About Outcome

The rationale for first-trimester sampling is not primarily diagnostic: it is that a negative result might exclude CMV-related embryopathy [8]. That proposition concerns sequelae. Every two-by-two table in this review has infection as its target condition, and no included report provides neurodevelopmental or audiological follow-up of children born after a negative test. The claim that motivates the test has never been evaluated.

The distinction is quantitatively material. By applying the estimate that approximately one third of fetuses infected after first-trimester maternal infection develop sequelae [2], then of 1000 women with a negative villus test approximately, 80 would still carry an infected fetus, while approximately 27 would be expected to have an affected child against approximately 42 per 1000 before the test. A negative result moves the absolute risk of an adverse outcome from roughly 4.2% to roughly 2.7%—a real but modest shift, and a poor foundation for an irreversible decision. This calculation assumes that the proportion of infected fetuses who develop sequelae does not depend on the villus result, which has not been studied. A negative result also does not obviate amniocentesis, which in the largest cohort was performed in every pregnancy irrespective of the villus result; the test, as practised, is additive to the existing pathway and adds the procedure-related risk of sampling from 11 to 14 weeks. The same asymmetry is already familiar from amniocentesis, after which children with a negative result still warrant follow-up [19].

### 4.2. Treatment Interposed Between the Index Test and Reference Standard

Valaciclovir was systematic at the contributing centre throughout enrolment. It reduced vertical transmission after early primary infection [6,7], and the observed prevalence of 12.7% was roughly a third of that expected without treatment [20]. Predictive values are therefore conditional on a treated prevalence and do not transport; the same sensitivity and specificity applied to a 35% prevalence would yield a negative predictive value near 76% rather than 92%—that is, a post-test probability of infection after a negative result of about 24% rather than 8%.

More fundamentally, an intervention acting between test and reference standard alters the target condition after the test has been performed. Some false negatives are pregnancies in which transmission had not yet occurred from 12 to 14 weeks; others are pregnancies in which it occurred despite treatment. Neither is a failure of the assay to detect virus that was present. The direction of the resulting bias cannot be resolved: preferential prevention of late transmission would bias sensitivity upward, while suppression of trophoblast viral replication without prevention of transmission would bias it downward. Both are plausible, and no contemporary untreated cohort exists against which to calibrate.

### 4.3. A Reference Standard That Partly Incorporates the Index Test

Retaining terminated pregnancies is methodologically right, but it forces a second verification route, and in the largest cohort, that route was placental tissue. CMV infects the placenta in the absence of transmission to the fetus, so a pregnancy classified as infected via a positive placenta may not have carried an infected fetus. The error is not random, as the index test measures viral DNA in the same compartment, so verification partly incorporates the test into its own reference standard, and misclassification correlated with the test instead of being independent of it.

The consequences depend on the villus result in the misclassified pregnancies. A pregnancy with an uninfected fetus but an infected placenta is counted as a true positive instead of a false positive if the villus test was positive, which inflates sensitivity, specificity, and positive predictive value; it is counted as a false negative instead of a true negative if the villus test was negative, which deflates sensitivity and, marginally, specificity and negative predictive value. Sampling of the same compartment makes the first scenario more likely, but villi taken at 11 to 14 weeks and placental tissue examined at termination are not the same specimen, so the second cannot be excluded. The number of terminations verified on placental tissue, cross-tabulated against the villus result, is not reported, and the net direction of the bias on sensitivity and specificity therefore cannot be determined. Two consequences are unambiguous: prevalence is overstated, and positive predictive value can only be overstated. The limitation does not alter the central finding that a negative result leaves a substantial proportion of infected fetuses undetected.

### 4.4. Concentration and Granularity of the Evidence

Of 340 villus samples, 330 were obtained, processed, and interpreted at one institution with one in-house assay under one antiviral policy, and all 42 infected pregnancies came from there. There was no external replication. The positivity criterion was carried over unchanged from the initial feasibility report and was therefore fixed before recruitment, which is a strength; what is missing is granularity, since the results were reported as positive or negative without the distribution of villus viral loads, so no alternative operating point can be examined.

### 4.5. Place of First-Trimester Diagnosis Among Current Approaches

Earlier diagnosis has potential benefits. For women who would consider termination, a positive result at 12 to 14 weeks brings forward counselling and, if chosen, a procedure at a gestational age at which it is technically simpler. For those continuing the pregnancy, it could allow targeted neurosonographic and magnetic resonance surveillance to be planned from the outset, and it could in principle identify earlier the fetuses who are candidates for in utero treatment with high-dose valaciclovir, which has so far been evaluated only from the second trimester [21]. A sufficiently sensitive negative result would also shorten the weeks of uncertainty that follow the diagnosis of maternal infection.

These benefits have to be weighed against the alternatives. Amniotic fluid PCR performed from 17 weeks onward and at least eight weeks after maternal infection is highly sensitive and specific, and a negative result after timely amniocentesis is considered to exclude long-term sequelae [5,22]; villus PCR achieved roughly half that sensitivity (Table 3) and, in the only cohort informative for sensitivity, did not replace amniocentesis, which was performed in every woman. Nor does a first-trimester result currently change antiviral management; valaciclovir is recommended from the diagnosis of periconceptional or first-trimester primary infection until the amniotic fluid result [5], and there is no evidence that starting fetal treatment at 12 weeks rather than 17 to 20 weeks improves outcomes. Against these limited gains stand the procedure-related risk of an additional invasive test and the unstudied question of whether sampling through an infected placenta at 11 to 14 weeks can itself facilitate transmission. Non-invasive first-trimester markers, such as trophoblast thickness and maternal blood and urine biomarkers, are being explored in the same population [17] but have not been validated against the neonatal reference standard. Maternal serology dates the infection and defines the prior risk, while fetal imaging and amniotic fluid viral load refine prognosis once infection is established [3,4]; villus PCR, as currently evaluated, adds information on infection status only, earlier but less completely than amniocentesis.

### 4.6. Strengths and Limitations

The review was registered prospectively, searched five databases without restriction, screened and extracted in duplicate, and applied an explicit rule for overlapping cohorts. Disentangling the nested reports from the largest centre is, in our view, its principal methodological contribution, since the nesting is not stated in any of them. This review suggests that the evidence base is too narrow to establish the sensitivity of the test, and its limitations are chiefly those of the literature. Only three independent cohorts met the eligibility criteria, and they are markedly unbalanced: one contributed 330 of the 340 samples and all 42 infected pregnancies, whereas the other two contributed four and six samples correspondingly, all true negatives, recruited between 1988 and 2000 with assays that would not be used today. The three datasets therefore do not replicate one another. The sensitivity estimate is in effect a single-centre estimate, obtained with one in-house assay, under universal valaciclovir prophylaxis and in a national referral population, and its transportability to other laboratories, to untreated women, and to settings in which primary infection is diagnosed less promptly is unknown. The specificity estimate is more robust, but the two older cohorts add little precision and are of limited applicability. Three datasets also preclude meta-analysis under our pre-specified rules, formal assessment of heterogeneity and investigation of small-study effects, and with very low certainty for both sensitivity and specificity; further studies are likely to change the estimates.

### 4.7. Implications for Practice and Research

Two conclusions should be distinguished. The diagnostic conclusion follows directly from the data: a negative villus PCR does not reliably exclude congenital infection. In the only cohort informative for sensitivity, it lowered the probability of infection from 12.7% to about 8% (Section 2.6), and it lowers the probability of an adverse neurodevelopmental outcome by even less; it does not exclude embryopathy, and no study has shown that it does. The clinical implication is an inference from that finding rather than a tested result. Because a negative result leaves a residual probability of infection of about one in twelve, and because no study has examined the outcome of children born after a negative test, villus PCR should not be used as a standalone basis for a decision about continuing or terminating a pregnancy, and amniocentesis at the recommended time remains necessary [5]. This inference rests on very low-certainty estimates from a single treated cohort and may change as further evidence accrues. A positive result is highly specific and may reasonably bring forward counselling and surveillance, provided both parties understand that it will still be confirmed by amniocentesis. Compared with amniotic fluid PCR from 17 weeks, first-trimester sampling enables information roughly five weeks earlier, at approximately half the sensitivity with the cost of an additional invasive procedure.

Future evaluation should be prospective and multi-centre; should report villus viral loads rather than a binary result; should verify terminated pregnancies on fetal rather than placental material; should report accuracy stratified by antiviral exposure, in centres with and without antiviral prophylaxis; and should follow children born after a negative test—the only design that can test the proposition on which the whole approach rests.

## 5. Conclusions

Direct detection of CMV in chorionic villi is highly specific but insensitive. The evidence derives almost entirely from one centre operating under universal antiviral prophylaxis, in which terminated pregnancies were verified on the same tissue the test samples, and no study has evaluated the outcome the test is intended to predict. A negative result does not reliably exclude congenital infection. On current, very-low-certainty evidence, villus PCR should not be used as a standalone basis for first-trimester decisions about continuing a pregnancy, and it does not replace amniocentesis.

## Figures and Tables

**Table 2:** Accuracy against the primary reference standard (congenital infection at birth).

Study	TP	FP	FN	TN	Sensitivity, % (95% CI)	Specificity, % (95% CI)	PPV, %	NPV, %	Prevalence, %
Bourgon 2026 [16]	17	2	25	286	40.5 (27.0–55.5)	99.3 (97.5–99.8)	89.5	92.0	12.7
Hogge 1993 [14]	0	0	0	4	not estimable	100 (51.0–100)	not estimable	100	0
Enders 2001 [15]	0	0	0	6	not estimable	100 (61.0–100)	not estimable	100	0

CI, confidence interval; FN, false negative; FP, false positive; NPV, negative predictive value; PPV, positive predictive value; TN, true negative; TP, true positive. Confidence intervals are Wilson score intervals. No pooled estimate was calculated per protocol. Certainty of evidence (GRADE) was very low for both sensitivity and specificity. In Bourgon 2026 [16], live-born infants were verified using neonatal urine and/or saliva within three weeks of birth and terminated pregnancies with placental tissue; the number of terminations and their distribution across the cells of the table are not reported (Section 4.3).

**Table 3:** Accuracy against amniotic fluid PCR as a secondary reference standard.

Report	n	TP	FP	FN	TN	Sensitivity, %	Specificity, %
Bourgon 2026 [16] (included)	330	14	5	15	296	48.3	98.3
Faure-Bardon 2021 [8] (companion)	37	3	0	3	31	50.0	100
Bourgon 2024 [18] (companion)	139	8	3	7	121	53.3	97.5

Companion reports are shown for completeness and are nested within the included report; they contribute no independent data.

**Table 4:** Consequences for 1000 women tested at the observed prevalence of 12.7%, applying the sensitivity and specificity of the largest cohort (Table 2) to a hypothetical population.

	Infected Fetus	Uninfected Fetus	Total
Positive villus test	51 (34–70)	6 (2–22)	57
Negative villus test	76 (57–93)	867 (851–871)	943
Total	127	873	1000

Figures are single-study estimates from Bourgon 2026 [16] rescaled to a hypothetical population of 1000 women with first-trimester primary infection and a pre-test probability of 12.7%, which was the prevalence observed under systematic valaciclovir; they are not pooled estimates. Infected fetuses (127) were multiplied by sensitivity (40.5%) and uninfected fetuses (873) by 1 − specificity (0.7%); ranges in parentheses apply the limits of the 95% CI. Of 943 women with a negative result, 76 (8.0%) still carried an infected fetus.

## Data Availability

All data analyzed in this review were derived from the published reports cited and are presented in the article and its Supplementary Materials. No new data were created.

## Supplementary Materials

The following supporting information can be downloaded at: https://www.mdpi.com/article/10.3390/medicina62101936/s1, Supplementary Table S1: PRISMA-DTA reporting checklist; Supplementary Table S2: Full search strategies for all five databases; Supplementary Table S3: Data extraction form, with the data extracted from the included studies; Supplementary Table S4: Reconstruction of the overlapping cohorts; Supplementary Table S5: QUADAS-2 signalling questions as tailored to this review, with judgements; Supplementary Table S6: Reports excluded at full-text assessment, with reasons.
