# Supplementary material for: Detection of Cytomegalovirus in Chorionic Villi for the Prenatal Diagnosis of Congenital Infection: A Systematic Review of Diagnostic Test Accuracy
Source: Medicina (Kaunas). 2026 Oct 8;62(10):1936. doi: 10.3390/medicina62101936 (PMC13707042; doi:10.3390/medicina62101936)
Supplement: Supplementary file 1 [file medicina-62-01936-s001.zip › medicina-4550941-supplementary.pdf]

Table S1. PRISMA-DTA reporting checklist.

Section	#	Item topic	Where reported
Title	1	Identification as a systematic review of diagnostic test accuracy	Title
Abstract	2	Structured summary (PRISMA-DTA for Abstracts)	Abstract
Introduction	3	Rationale	Section 1, paragraphs 1–3
	D1	Clinical role of the index test	Section 1, paragraphs 2–3 (replacement test brought forward to 11–14 weeks, ahead of amniotic-fluid PCR)
	4	Objectives	Section 1, last paragraph
Methods	5	Protocol and registration	Section 2 (PROSPERO CRD420261482510)
	6	Eligibility criteria	Section 2.1
	7	Information sources	Section 2.2
	8	Search	Section 2.2; Supplementary Table S2
	9	Study selection	Section 2.3
	10	Data collection process	Section 2.3; Supplementary Table S3
	11	Definitions for data extraction	Section 2.1; Supplementary Table S3
	12	Risk of bias and applicability	Section 2.5; Supplementary Table S5
	13	Diagnostic accuracy measures	Section 2.6
	14	Synthesis of results	Section 2.6 (pre-specified thresholds; meta-analysis not feasible)
	D2	Meta-analysis	Section 2.6 (bivariate model not applicable; no pooling)
	16	Additional analyses	Section 2.6 (rescaling to 1000 women; post-test probability; prevalence scenario at 35%); Deeks' test not performed
Results	17	Study selection	Section 3.1; Figure 1; Supplementary Table S6
	18	Study characteristics	Section 3.2; Table 1; Appendices S3–S4
	19	Risk of bias and applicability	Section 3.4; Figure 2; Supplementary Table S5
	20	Results of individual studies	Section 3.3; Tables 2 and 3
	21	Synthesis of results	Section 3.3; Table 4 (no pooled estimate)
	23	Additional analysis	Section 3.3; Section 3.5 (GRADE)
Discussion	24	Summary	Section 4, opening paragraph; Sections 4.1–4.5
	25	Limitations	Sections 4.2–4.4 and 4.6
	26	Conclusions	Section 4.7; Section 5
Other	27	Funding	Funding statement (no external funding)

Checklist based on McInnes et al., JAMA 2018;319:388-396 (PRISMA-DTA). Item numbering follows PRISMA-DTA, in which items 15 and 22 of PRISMA are not used and items D1 and D2 are specific to diagnostic test accuracy. Item topics are abbreviated; the full item wording is available at www.prisma-statement.org.(Accessed on 30 August 2026) PCR, polymerase chain reaction.

Table S2. Full search strategies for all five databases.

**MEDLINE via PubMed**		
**#**	**Query**	**Records**
#A	"Cytomegalovirus"[Mesh] OR "Cytomegalovirus Infections"[Mesh] OR cytomegalovir*[tiab] OR CMV[tiab] OR HCMV[tiab]	
#B	"Chorionic Villi Sampling"[Mesh] OR "Chorionic Villi"[Mesh] OR "chorionic vill*"[tiab] OR "chorion vill*"[tiab] OR trophoblast*[tiab] OR "placental biops*"[tiab]	
#C	#A AND #B	253
**Embase (Embase.com)**		
**#**	**Query**	**Records**
#1	'cytomegalovirus'/exp OR 'cytomegalovirus infection'/exp OR cytomegalovir*:ti,ab,kw OR cmv:ti,ab,kw OR hcmv:ti,ab,kw	
#2	'chorion villus sampling'/exp OR 'chorion villus'/exp OR (chorion* NEAR/2 vill*):ti,ab,kw OR trophoblast*:ti,ab,kw OR (placenta* NEAR/2 biops*):ti,ab,kw	
#3	#1 AND #2	420
**Scheme**		
**#**	**Query**	**Records**
#1	TITLE-ABS-KEY (cytomegalovir* OR cmv OR hcmv) AND TITLE-ABS-KEY ("chorionic vill*" OR "chorion vill*" OR trophoblast* OR "placental biops*")	408
**Web of Science Core Collection**		
**#**	**Query**	**Records**
#1	TS=(cytomegalovir* OR CMV OR HCMV)	
#2	TS=("chorionic vill*" OR "chorion vill*" OR trophoblast* OR "placental biops*")	
#3	#1 AND #2	322
**Cochrane CENTRAL (Cochrane Library)**		
**#**	**Query**	**Records**
#1	[mh Cytomegalovirus] OR [mh "Cytomegalovirus Infections"] OR (cytomegalovir* OR CMV OR HCMV):ti,ab,kw	
#2	[mh "Chorionic Villi Sampling"] OR [mh "Chorionic Villi"] OR ("chorionic vill*" OR "chorion vill*" OR trophoblast* OR "placental biops*"):ti,ab,kw	
#3	#1 AND #2 (trials)	2

All databases were searched on 19 August 2026, from inception, without language, date or publication-type restrictions. The strategy combined two concepts – cytomegalovirus (A) and chorionic villi or placental sampling (B) – with AND. No filter for diagnostic accuracy studies was applied. The search strategy was not peer reviewed by an external information specialist (PRESS); this is recorded as a deviation from protocol (Section 2.2). Records were exported to Rayyan and deduplicated (1405 records, 701 duplicates, 704 unique records).Four control queries were run in PubMed to test

whether additional terms would have retrieved further records: adding CVS(chorionic villus sampling)[tiab]; adding "placental tissue"[tiab]; adding both; and a known-item check for the included and companion reports. None retrieved any record not already captured by the final strategy (0 additional records each). Reference lists of included studies and relevant reviews were screened; no additional records were identified by citation searching or other methods.

Table S3. Data extraction form, with the data extracted from the included studies.

Field	Hogge 1993 [14]	Enders 2001 [15]	Bourgon 2026 [16]
Citation	Prenat. Diagn. 1993;13:131–136	Prenat. Diagn. 2001;21:362–377	Am. J. Obstet. Gynecol. 2026;234:1130–1141
Companion reports (E9)	none	none	Faure-Bardon 2021; Bourgon 2024; Bourgon 2025 (Supplementary Table S4)
Country / centre	United States (single centre)	Stuttgart, Germany (single laboratory)	Necker-Enfants Malades, Paris, France (national referral centre)
Design	case series	retrospective cohort	prospective cohort, consecutive enrolment
Enrolment period	not stated	1988 – February 2000	October 2019 – December 2024
Population	pregnancies with maternal CMV infection	189 pregnancies with primary infection and known outcome	periconceptional or first-trimester primary infection; CVS and amniocentesis in all
Parent cohort / villus samples	8 / 4	189 / 6	330 / 330
GA at villus sampling	10–16 weeks	10–18 weeks	11–14 weeks
Sampling route	1 transcervical (10 weeks), 3 transabdominal (second trimester)	not stated	transabdominal CVS
Index test	PCR on villi	PCR and virus isolation in culture	quantitative in-house CMV PCR on villi
Positivity threshold	not stated	not stated	as in feasibility report (Faure-Bardon 2021); pre-specified; reported as binary
Primary reference standard	neonatal urine culture	neonatal urine within 2 weeks	neonatal urine and/or saliva within 3 weeks; placental tissue after TOP
Secondary reference standard (AF PCR)	–	–	AF PCR in all (Table 3)
Antiviral exposure between index test and reference standard	none	none	valaciclovir, systematic throughout enrolment
Pregnancies not reaching live birth	–	not reported	retained in denominator; verified on placenta; number not reported
2×2 vs primary reference (TP/FP/FN/TN)	0/0/0/4	0/0/0/6	17/2/25/286

Field	Hogge 1993 [14]	Enders 2001 [15]	Bourgon 2026 [16]
2×2 vs AF PCR (TP/FP/FN/TN)	–	–	14/5/15/296
Per-case data	yes	no ("data not shown")	no
Notes	3 of 4 samples were second-trimester placental biopsies (late CVS)	villi available in 6 of 189 pregnancies; risk group not reported	abstract misattributes AF-based estimates; extracted from tables

Data were extracted in duplicate (I.M., M.D.) into a piloted Excel spreadsheet; disagreements were resolved by a third reviewer (G.R.). Where two-by-two counts were not presented directly, they were reconstructed from numerators, denominators and accuracy statistics and cross-checked against the text. For Bourgon 2026, counts were extracted from the results text and tables only, because the abstract attributes to the prediction of congenital infection the pair of estimates that the tables report against amniotic fluid. CMV, cytomegalovirus; AF, amniotic fluid; PCR, Polymerase chain reaction; TOP, termination of pregnancy.

Table S4. Reconstruction of the overlapping cohorts.

Report	Type	Enrolment window	*n* with CVS	Focus / index test	Reference standard	Two-by-two (TP/FP/FN/TN)	Status
Faure-Bardon 2021, Ultrasound Obstet. Gynecol. 57:568–572 [8]	prospective feasibility study	Oct 2019 – Oct 2020	37	CVS PCR	AF PCR; neonatal outcome known in 24/37	vs AF 3/0/3/31; vs neonatal 3/0/2/19	E9 – nested in Bourgon 2024 and 2026
Bourgon 2024, Ultrasound Obstet. Gynecol. 64(S1):129, EP04.01 [18]	congress abstract	Oct 2019 – Mar 2023	139	CVS PCR	AF PCR	vs AF 8/3/7/121	E9 – nested in Bourgon 2026
Bourgon 2025, Ultrasound Obstet. Gynecol. 66:598–605 [17]	cohort	Oct 2019 – May 2024	127	maximum trophoblast thickness and maternal biomarkers; CVS PCR as comparator	CVS and AF PCR	CVS/AF cross-classification: 6 (+/+), 3 (+/−), 6 (−/+), 112 (−/−)	E9 – subset of Bourgon 2026 defined by availability of 3D volumes
Bourgon 2026, Am. J. Obstet. Gynecol. 234:1130–1141 [16]	prospective cohort	Oct 2019 – Dec 2024	330	CVS PCR	neonatal urine/saliva (placenta after TOP); AF PCR	vs neonatal 17/2/25/286; vs AF 14/5/15/296	Included – primary data source
Faure-Bardon 2021, Ultrasound Obstet. Gynecol. 58:576–581 [7]	cohort (valaciclovir)	2009 – 2020	10 (previously published)	secondary prevention with valaciclovir	–	–	E2 – index test not evaluated; confirms systematic valaciclovir from Oct 2019

Four reports from Necker-Enfants Malades Hospital describe CVS-based testing in women with periconceptional or first-trimester primary infection enrolled from October 2019 onwards, and a fifth report from the same centre refers to cases already published. None of them states the overlap explicitly. Nesting was reconstructed from enrolment windows, sample sizes, eligibility criteria and cross-references (Section 2.4). The report with the largest sample and most complete verification (Bourgon 2026) was retained as the primary data source; the others were treated as companion publications (E9) contributing no two-by-two data. Nesting：Faure-Bardon 2021 (October 2019 – October 2020; n = 37) ⊂ Bourgon 2024 (to March 2023; n = 139) ⊂ Bourgon 2026 (to December 2024; n = 330). Bourgon 2025 (to May 2024; n = 127) is a separate subset of the same enrolment defined by the availability of three-dimensional placental volumes. Presenting the four Necker reports as independent would have inflated the apparent evidence base fourfold and would have counted the same infected pregnancies up to four times. Observations relevant to interpretation: (i) valaciclovir was given systematically from the diagnosis of maternal infection throughout the Necker enrolment period, as documented in Faure-Bardon 2021 (Ultrasound Obstet. Gynecol. 58); (ii) Faure-Bardon 2021 (Ultrasound Obstet. Gynecol. 57) is the only Necker report in which a relevant proportion of women (9 of 37) did not receive preventive valaciclovir; (iii) the positivity criterion of Bourgon 2026 refers back to the assay described in Faure-Bardon 2021 and was therefore fixed before most of the recruitment; (iv) in Bourgon 2025, amniocentesis was performed in all women after CVS, irrespective of the villus result; (v) recruitment increased from 139 women in the first 41 months to 330 by the end of the following 21 months, which could not be explained from the published reports. Abbreviations: AF, amniotic fluid; CVS, chorionic villus sampling; FN, false negative; FP, false positive; GA, gestational age; NPV, negative predictive value; PCR, polymerase chain reaction; TN, true negative; TOP, termination of pregnancy; TP, true positive.

Table S5. QUADAS-2 signalling questions as tailored to this review, with judgements.

Signalling questions	
**Domain**	**Signalling questions**
1. Patient selection	1.1 Was a consecutive or random sample of women with primary maternal infection enrolled? 1.2 Was a case-control design avoided? 1.3 Did the study avoid inappropriate exclusions (e.g., selection of the villus-tested subset by availability of tissue)? *1.4 Was primary infection documented virologically, with a dated seroconversion window?
2. Index test	2.1 Were villus results interpreted without knowledge of the reference standard? 2.2 If a threshold was used, was it pre-specified? *2.3 Was the assay (target, extraction, quantification) described sufficiently to be reproduced? *2.4 Were villus results reported for every sampled pregnancy?
3. Reference standard	3.1 Is the reference standard likely to classify congenital infection correctly (neonatal urine and/or saliva within three weeks of birth)? 3.2 Were reference results interpreted without knowledge of the villus result? *3.3 For pregnancies not reaching live birth, was the verification pre-defined, and was it performed on fetal rather than placental material (placental material partly incorporates the index test)?
4. Flow and timing	4.1 Was there an appropriate interval between index test and reference standard? *4.2 Was antiviral treatment (valaciclovir or hyperimmune globulin) avoided between index test and reference standard, or were results reported by exposure? 4.3 Did all women receive a reference standard? 4.4 Did all women receive the same reference standard? 4.5 Were all women included in the analysis?
Applicability	Patients: do the women included match the review question (first-trimester primary infection, first-trimester sampling in ongoing pregnancy, contemporary care)? Index test: does the test match the review question (contemporary PCR on villi at 11-14 weeks)? Reference standard: does the target condition match (congenital infection at birth)?
**Consensus judgements (Figure 2)**	

Domain	Hogge 1993	Enders 2001	Bourgon 2026
Patient selection – risk	High: 4 of 8 pregnancies tested, subset selection not described	High: villi available in 6 of 189 pregnancies; selection and risk group not reported	Low: consecutive enrolment, documented primary infection
Index test – risk	Unclear: blinding and interpretation criteria not reported	High: combined PCR and culture; assay and positivity criteria not described; results not reported by case	Low: positivity criterion pre-specified (feasibility report); results reported for all
Reference standard – risk	Low: neonatal urine culture	Low: neonatal urine within 2 weeks	High: terminated pregnancies verified on placental tissue (Section 4.3)
Flow and timing – risk	Low: all four verified with the same reference standard	Unclear: interval and handling of non-live births not reported	High: valaciclovir systematic between index test and reference standard; two verification routes
Applicability – patients	High concern: mostly second-trimester sampling; small undocumented subset	High concern: sampling up to 18 weeks; risk group not reported	High concern: universal valaciclovir prophylaxis; national referral population
Applicability – index test	High concern: early non-quantitative PCR	High concern: assays of 1988-2000, including culture	Low concern
Applicability – reference standard	Low concern	Low concern	Low concern

QUADAS-2 (Whiting et al., Ann. Intern. Med. 2011;155:529-536) was applied independently by two reviewers (I.M., M.D.), with disagreements resolved by a third (G.R.). Standard signalling questions were retained; review-specific questions (marked *) were added to address verification of pregnancies not reaching live birth and antiviral treatment between index test and reference standard. QUADAS-C was not applicable. Answers: Y, yes; N, no; U, unclear.PCR, polymerase chain reaction.

Table S6. Reports excluded at full-text assessment, with reasons.

A. Exclusions by category		
**Code**	**Reason for exclusion**	***n***
E1	definition as in protocol	50
E2	Index test not evaluated (no direct CMV testing of chorionic villi)	16
E3	Villi not obtained by prenatal sampling in an ongoing pregnancy (e.g., obtained at termination)	14
E4	No eligible reference standard	0
E5	Two-by-two data not extractable or reconstructable	0
E6	Fewer than four villus samples	0
E7	definition as in protocol	10
E8	definition as in protocol	5
E9	Companion publication of an included cohort (overlapping population)	3

E10	Full text not retrievable	0
	Total	98
**B. Reports that might appear eligible, with reasons for exclusion**		
**Report**	**Reason**	**Code**
Dong ZW et al. Detection of congenital cytomegalovirus infection by using chorionic villi of the early pregnancy and polymerase chain reaction. Int. J. Gynaecol. Obstet. 1994;44:229–231.	Villi obtained from terminations of pregnancy, not by prenatal sampling in ongoing pregnancies	E3
Enders G et al. The value of CMV IgG avidity and immunoblot for timing the onset of primary CMV infection in pregnancy. Prenat. Diagn. 2017;37:389–398.	Amniotic fluid and fetal blood only; placental tissue used only to confirm infection after termination	E2
Faure-Bardon V et al. Secondary prevention of congenital cytomegalovirus infection with valacyclovir following maternal primary infection in early pregnancy. Ultrasound Obstet. Gynecol. 2021;58:576–581.	Evaluates valaciclovir, not the index test; villus cases previously published (Supplementary Table S4)	E2
Faure-Bardon V et al. First-trimester diagnosis of congenital cytomegalovirus infection … by PCR on chorionic villi obtained by CVS. Ultrasound Obstet. Gynecol. 2021;57:568–572.	Nested in Bourgon 2026 (Supplementary Table S4)	E9
Bourgon N et al. EP04.01: First trimester diagnosis of cytomegalovirus trophoblastic infection using CMV-PCR. Ultrasound Obstet. Gynecol. 2024;64(S1):129.	Nested in Bourgon 2026 (Supplementary Table S4)	E9
Bourgon N et al. Non-invasive prenatal diagnosis of congenital cytomegalovirus infection using maximum trophoblast thickness and biomarkers in maternal blood and urine in first trimester. Ultrasound Obstet. Gynecol. 2025;66:598–605.	Nested in Bourgon 2026 (Supplementary Table S4)	E9

Of 101 reports assessed on the full text, 98 were excluded. Ineligibility was evident on first inspection in 85; the remaining 16 required detailed reading, after which 13 were excluded and 3 included (Section 3.1). One reason was assigned per report, hierarchically, with companion publication (E9) taking precedence. CMV, cytomegalovirus; PCR, polymerase chain reaction; CVS, chorionic villus sampling.
